# Impact of SARS-CoV2 infection on anti-apolipoprotein A-1 IgG response in inflammatory rheumatic diseases

**DOI:** 10.3389/fimmu.2023.1154058

**Published:** 2023-05-10

**Authors:** Celine Lamacchia, Denis Mongin, Catherine Juillard, Paola Antinori-Malaspina, Cem Gabay, Axel Finckh, Sabrina Pagano, Nicolas Vuilleumier

**Affiliations:** ^1^ Division of Rheumatology, Geneva University Hospital and Faculty of Medicine, University of Geneva, Geneva, Switzerland; ^2^ Division of Laboratory Medicine, Department of Diagnostics and of Medical Specialties, Geneva University Hospitals and Geneva University, Geneva, Switzerland; ^3^ Geneva Center for Inflammation Research (GCIR), University of Geneva, Geneva, Switzerland

**Keywords:** anti-apolipoprotein A-1 IgG, autoantibodies, severe acute respiratory syndrome coronavirus 2, inflammatory rheumatic diseases, autoimmunity

## Abstract

**Objectives:**

To investigate the impact of severe acute respiratory syndrome coronavirus 2 (SARS-CoV2) infection on anti-apolipoprotein A-1 IgG (AAA1) humoral response in immunosuppressed inflammatory rheumatic diseases (IRD) patients.

**Methods:**

This is a nested cohort study from the prospective Swiss Clinical Quality Management registry. A total of 368 IRD patients for which serum samples were available before and after the SARS-CoV2 pandemic were included. Autoantibodies against ApoA-1 (AAA1) and its c-terminal region (AF3L1) were measured in both samples. The exposure of interest was anti-SARS-CoV2 spike subunit 1 (S1) seropositivity measured in the second sample. The effect of SARS-CoV2 infection (anti-S1 seropositivity) on becoming AAA1 or AF3L1 positive and on the change of AAA1 or AF3L1 optical density (OD) between the two samples was tested with multivariable regressions.

**Results:**

There were 12 out of 368 IRD patients who were seroconverted against S1. The proportion of patients becoming AF3L1 seropositive was significantly higher in anti-S1-positive patients, compared with anti-S1-negative patients (66.7% versus 21.6%, p = 0.001). Adjusted logistic regression analyses indicated that anti-S1 seroconversion was associated with a sevenfold increased risk of AFL1 seropositivity (odds ratio: 7.4, 95% confidence interval (95% CI): 2.1–25.9) and predicted median increase in AF3L1 OD values (+0.17, 95% CI: 0.08–0.26).

**Conclusions:**

SARS-CoV2 infection is associated with a marked humoral response against the immunodominant c-terminal region of ApoA-1 in IRD patients. The possible clinical impact of AAA1 and AF3L1 antibodies on disease progression, cardiovascular complications, or long COVID syndrome deserves future investigations.

## Highlights

- SARS-CoV2 infection is associated with a marked humoral response against the immunodominant c-terminal region of ApoA-1 in inflammatory rheumatic diseases (IRD) patients.
- These results are relevant because it confirms that exposure to a viral agent may lead to the development of autoimmunity in IRD patients.
- The impact of such biological signature on IRD disease progression and complications such as cardiovascular events warrants further dedicated studies.

## Introduction

Apolipoprotein A-1 (ApoA-1) is one of the main proteins of high-density lipoprotein (HDL) particles and expresses its atheroprotective properties through pleiotropic effects. The literature supports that humoral autoimmunity against ApoA-1 is a rather frequent phenomenon associated with poorer clinical outcomes, disease activity, and subclinical atherosclerosis in various settings, including autoimmune diseases, such as rheumatoid arthritis (RA) and systemic lupus erythematous (SLE), and cardiovascular (CV) diseases, and in the general population (1–4). Anti-ApoA-1 IgG autoantibodies (AAA1) act as pro-atherogenic molecules through Toll-like receptor (TLR)-2 and TLR-4, promoting low-grade inflammation and intracellular lipid accumulation culminating into foam cell formation (5).

Viruses are known to be important environmental factors that may contribute to the development of autoimmunity and autoimmune diseases (6). Like many viral infections, severe acute respiratory syndrome coronavirus 2 (SARS-CoV2) infection can lead to development of broad autoantibody responses (7). Such phenomenon is partly explained by the presence of sequence/structural homologies between SARS-CoV2 immunoreactive epitopes, its receptor-binding domain (RBD), and numerous host self-proteins (8, 9). Recently, Pagano et al. reported common linear epitopes between SARS-CoV2 and the c-terminal part of ApoA-1 and TLR-2. The authors have reported an association between anti-SARS-CoV2 and AAA1 humoral responses in a case–control study, both in a prospective intensive care unit (ICU) cohort and in a general population cohort (10). Interestingly, another longitudinal prospective study indicated that the SARS-CoV2-induced AAA1 response could concern up to 90% of immunocompetent infected individuals at 3 months and independently predict symptom persistence at 1 year (11).

So far, RA is the main inflammatory rheumatic disease (IRD) known to be associated with an increased prevalence of AAA1 seropositivity, which is strongly associated with an increased incidence of CV events, prior to the SARS-CoV2 pandemic (12). Therefore, we hypothesised that SARS-CoV2 infection would affect the AAA1 response in IRDs and investigated the clinical and biological determinants of such response using 368 IRD patients enrolled in the Swiss Clinical Quality Management (SCQM) registry during the pre-pandemic SARS-CoV2 period and resampled during the pre-/post-pandemic period.

## Methods

### Study population

This is a nested cohort study from a prospective, longitudinal, cohort of IRDs patients, the Swiss Clinical Quality Management registry (SCQM, www.SCQM.ch). The SCQM registry was founded in 1997 with the support of Swiss regulatory authorities and aims at continuously improving the quality of treatment of RA, axial spondyloarthritis (AxSpA), and psoriatic arthritis (PsA). Unlike many other European registries, most patients are enrolled by private office-based rheumatologists (60%), providing a genuine population-based sample of RA patients in Switzerland. The data for this study were extracted from the SCQM registry on 01/12/2021. The study protocol was approved by the SCQM Biobank Scientific Advisory Board and the SCQM Foundation Board and the local ethics committee of the University Hospital of Geneva (PB_2018-00317). All participants gave informed consent before enrolment, in accordance with the Declaration of Helsinki.

### Samples and biochemical analyses

We used the subgroup of 368 patients from the SCQM registry for which serum samples were available before (sample 1) and after the COVID-19 outbreak (sample 2). The initial sample collection (sample 1) took place several years prior to the SARS-CoV2 pandemic; the second sample collection (sample 2) occurred between 11/08/2020 and 02/02/2021. Serum samples were processed and stored at −80°C until analysis.

Sample size calculation based on the results in the general population (considering 15% *vs*. 40% of seroconversion (10)) indicated that 30 participants with SARS-CoV2 would be enough to detect such effect with a power of 85%.

AAA1 autoantibodies and those directed against the c-terminal part of ApoA-1 (AF3L1) were measured in both samples using extensively validated in-house ELISA protocol (13, 14). Seropositivity cut-offs for AAA1 and AF3L1 were prospectively defined and set at an optical density (OD) measured at 405 nm (OD405) >0.64 and >0.5, respectively. These cut-offs correspond to the 97.5th percentile of AAA1 and AF3L1 levels obtained from healthy blood donors (13, 14).

High-density lipoprotein (HDL), non-HDL, and low-density lipoprotein (LDL) cholesterol levels were measured in sample 1 by standard chemistry assays (Roche 8000/H cobas), whereas LDL cholesterol values were calculated using the Friedewald formula.

In sample 2, quantitative SARS-CoV2 antibody testing was performed using the Roche Elecsys anti-SARS-CoV2 spike subunit 1 (S1) assay with an anti-S1 seropositivity cut-off set at 0.8 U/ml according to the manufacturer’s instruction.

### Outcome

The primary outcome of this analysis was a seroconversion of AAA1 or AF3L1 between sample 1 and sample 2. This binary outcome was coded 1 for patients AAA1 negative in sample 1 and positive in sample 2, and coded 0 otherwise (stayed positive, stayed negative, or switched from positive to negative). The secondary outcome was the change of OD of AAA1 and AF3L1 between sample 1 and sample 2.

### Exposure

The exposure of interest was a SARS-CoV2 infection as established by a positive anti-S1 seropositivity in sample 2. Potential confounders considered were the type of immune-mediated disease, the disease duration at sample 2, the age at sample 2, the sex of the patients, the time elapsed between the collection of the two samples, the cumulated dose of glucocorticoid (GC) taken, and the duration on biological disease-modifying anti-rheumatic drugs (bDMARDs) during this period.

### Statistical analysis

We had no missing data in the studied data set. Differences in proportion of categorical variables between anti-S1 positive and anti-S1 negative were tested with the chi-square test. Differences for continuous variables were tested with the T-test, and with the Wilcoxon test for the variables with a non-normal distribution. Confidence intervals of binomial proportion were calculated using the Wilson method. Continuous variables were presented as means and standard deviations (SD), or as medians and interquartile ranges (IQR) when appropriate.

The effect of anti-S1 seropositivity on AAA1 or AF3L1 seroconversion in sample 2 was tested using multivariable logistic regression models with a binomial distribution. The effect of anti-S1 seropositivity on the change of AAA1 or AF3L1 OD was assessed with multivariable linear regression. Results are expressed with 95% confidence intervals (95% CI). p values < 0.05 were considered as significant. All analyses were performed with R software (V.4.0.4).

## Results

### Population description

The patients’ characteristics are presented in **Table 1**. Among the 368 patients considered in this study, 43% had RA, 36% AxSpA, and 21% PsA. Patients were predominantly women (63%), with a mean age of 54 years. Across the IRD spectrum, the baseline prevalence of AAA1 and AF3L1 seropositivity was respectively 11.2% [95% CI: 7.2%, 17.0%] and 4.3% [95% CI:2.1%, 8.7%] in RA, 18.4% [95% CI:11.3%, 28.6%] and 3.9% [95% CI:1.4%, 11.0%] in AxSpA, and 16.8% [95% CI: 11.4%, 24.1%] and 3.1% [95% CI:1.2%, 7.6%] in PsA. No significant difference was observed regarding the prevalence of AAA1 and AF3L1 seropositivity between these three diseases. Median disease duration was 12 years, with no significant difference across the three IRD subgroups. The median time difference between sample 1 and sample 2 was 6 years [IQR: 4-8]. During this period of time, 12 patients seroconverted against S1, 93 patients became AAA1 positive, and 85 became AF3L1 positive. Between samples 1 and 2, the optical densities of these two antibodies increased by 0.2 OD units. If 97.2% of the second sample collection (sample 2) occurred before the start of the SARS-CoV2 vaccination campaign in Switzerland (28/12/2020), 100% (12/12) of the anti-S1 positive patients were unvaccinated, implying a COVID-19-induced S1 seroconversion.

**Table 1:** Sociodemographic characteristics and serum sample results of the ensemble of the patients considered (overall), and per group of anti-S1 positivity.

	Overall	Anti-S1 negative	Anti-S1 positive	p
Patient characteristics (at sample 2)				
N patients	368	356	12	
Sex = male (%)	137 (37.2)	132 (37.1)	5 (41.7)	0.984
Age (mean (SD))	53.80 (12.32)	53.74 (12.42)	55.46 (9.07)	0.636
Disease (%)				0.931
AxSpA	131 (35.6)	127 (35.7)	4 (33.3)	
PsA	76 (20.7)	73 (20.5)	3 (25.0)	
RA	161 (43.8)	156 (43.8)	5 (41.7)	
Disease duration (years) (median [IQR])	12.78 [8.04, 19.04]	12.77 [7.95, 18.75]	16.14 [10.96, 26.72]	0.149
Pre-pandemic serum characteristics (at sample 1)				
AAA1 IgG positive (%)	54 (14.7)	50 (14.0)	4 (33.3)	0.149
AF3L1 IgG positive (%)	14 (3.8)	13 (3.7)	1 (8.3)	0.947
Total cholesterol (mean (SD))	5.29 (1.21)	5.29 (1.17)	5.24 (2.15)	0.892
HDL cholesterol (mean (SD))	1.51 (0.43)	1.51 (0.42)	1.51 (0.63)	0.986
Non-HDL cholesterol (mean (SD))	3.78 (1.15)	3.78 (1.13)	3.73 (1.72)	0.881
LDL cholesterol (mean (SD))	1.44 (0.81)	1.43 (0.77)	1.74 (1.71)	0.194
Difference between post-pandemic and pre-pandemic samples				
Delay between samples in years (median [IQR])	6.05 [4.25, 7.88]	5.98 [4.22, 7.86]	7.34 [5.28, 8.35]	0.244
Became AAA1 positive (%)	93 (25.3)	87 (24.4)	6 (50.0)	0.096
AAA1 OD difference (mean (SD))	0.23 (0.25)	0.23 (0.24)	0.31 (0.40)	0.276
Became AF3L1 positive (%)	85 (23.1)	77 (21.6)	8 (66.7)	**0.001**
AF3L1 OD difference (mean (SD))	0.21 (0.16)	0.20 (0.16)	0.37 (0.15)	**<0.001**
Under GC treatment between sample 1 and sample 2	56 (15.2)	54 (15.2)	2 (16.7)	1
Cumulative GC dose (in g) of those under GC treatment (median [IQR])	2.37 [0.89, 7.83]	2.37 [0.88, 8.02]	2.67 [1.96, 3.38]	0.825
Cumulative years of bDMARD treatment (median [IQR])	4.30 [1.61, 6.82]	4.27 [1.59, 6.73]	6.76 [4.00, 7.83]	0.076

The sociodemographic characteristics at sample 2 collection are the sex, the age, the disease (RA, rheumatoid arthritis; PsA, psoriatic arthritis; AxSpA, axial spondyloarthritis), and the disease duration. The serum baseline characteristics are the percentage of anti-ApoA-1 (AAA1) IgG and anti-F3L1 (AF3L1) IgG positive, titres (mmol/L) of total, high-density lipoprotein (HDL), non-HDL, and low-density lipoprotein (LDL) cholesterol. The sample difference provides the delay in years between sample 1 and sample 2, the number of patients who became AAA1 or AF3L1 positive and the associated change in optical density (OD), the number of patients under glucocorticoid (GC) treatment between sample 1 and sample 2, the cumulated dose of GC of those under GC, and the cumulative time under biological disease-modifying antirheumatic drugs (bDMARDs) between sample 1 and sample 2. The delay between samples, the number of years under bDMARDs, the disease duration, and the GC dose cumulated have a non-normal distribution: the comparison between the groups are performed with the Wilcoxon test. Bold *p*-values of 0.05 or less were considered statistically significant.

**Figure 1:**
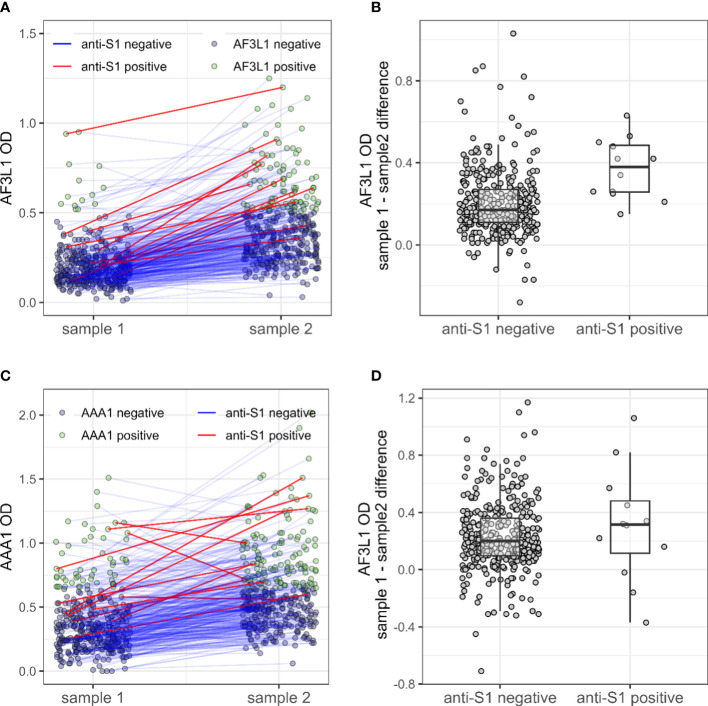
Anti-ApoA-1 IgG, anti-F3L1 IgG, and anti-S1 IgG status in patients with inflammatory rheumatic diseases during the pre- and pre-/post SARS-CoV2 era. Anti-F3L1 (AF3L1) IgG **(A)** and anti-ApoA-1 (AAA1) IgG **(C)** optical density (OD) measured in pre-pandemic sample (sample 1) and pre-/post-pandemic samples (sample 2), with a line linking the two measures for the same patient. The line is coloured in red for patients with a positive anti-S1 measure in sample 2. The two right panels display the OD difference between sample 2 and sample 1 for AF3L1 IgG **(B)** and AAA1 IgG **(D)**, for patients anti-S1 positive and the others.

Patients who seroconverted against SARS-CoV2 displayed a higher AF3L1 seropositivity rate and median AF3L1 OD values, compared with uninfected patients by COVID-19, and a similar trend was observed for AAA1 median OD values (**Table 1**, **Figure 1**).

### Multivariable regression

Logistic regression analyses demonstrated that male gender was the only significant predictor of AAA1 seroconversion (OR 2.23; 95% CI: 1.30–3.82; **Table 2**). SARS-CoV2 infection increased the risk of AF3L1 seropositivity by sevenfold (OR: 7.4; 95% CI: 2.14–25.8) and was associated with an increase of AF3L1 values by 0.18 OD (95% CI: 0.08–0.27), as was disease duration (0.17; 95% CI: 0.081–0.26; **Table 2**).

**Table 2:** Result of the logistic regression.

	AAA1 IgG				AF3L1 IgG			
	Odds ratio of becoming AAA1 IgG positive	p	Estimate on AAA1 OD difference	p	Odds ratio of becoming AF3L1 IgG positive	p	Estimate on AF3L1 OD difference	p
**(Intercept)**	0.14 [0.034, 0.55]	0.005	0.18 [0.039, 0.31]	0.01	0.33 [0.081, 1.31]	0.12	0.21 [0.12, 0.30]	<0.001
**Anti-S1 positivity**	2.86 [0.88, 9.31]	0.08	0.092 [-0.048, 0.23]	0.20	7.46 [2.15, 25.92]	0.002	0.18 [0.085, 0.27]	<0.001
**Sex male**	2.23 [1.30, 3.82]	0.003	-0.022 [-0.077, 0.034]	0.45	0.96 [0.55, 1.69]	0.90	-0.0044 [-0.04, 0.032]	0.81
**Age**	1.01 [0.99, 1.04]	0.24	0.00091 [-0.0014, 0.0032]	0.43	0.997 [0.97, 1.02]	0.81	-0.00031 [-0.0018, 0.0012]	0.68
Disease (reference RA)								
**AxSpa**	0.67 [0.36, 1.24]	0.20	0.024 [-0.039, 0.087]	0.46	0.78 [0.41, 1.49]	0.46	0.0085 [-0.032, 0.049]	0.68
**PsA**	0.54 [0.26, 1.11]	0.09	-0.027 [-0.099, 0.045]	0.45	1.29 [0.65, 2.59]	0.47	0.013 [-0.033, 0.06]	0.57
**Years on bDMARDS**	1.04 [0.93, 1.16]	0.49	-0.0052 [-0.016, 0.0059]	0.36	1.01 [0.90, 1.13]	0.90	-0.0051 [-0.012, 0.0021]	0.16
**Cumulated GC dose**	0.94 [0.85, 1.04]	0.26	-0.0039 [-0.012, 0.0041]	0.35	0.95 [0.85, 1.06]	0.32	-0.00054 [-0.0057, 0.0047]	0.84
**Time interval between sample 1 and sample 2**	0.98 [0.85, 1.12]	0.77	0.011 [-0.0026, 0.025]	0.11	1.03 [0.89, 1.18]	0.70	0.0054 [-0.0035, 0.014]	0.23
**Disease duration**	1.005 [0.98, 1.03]	0.75	0.082 [-0.061, 0.22]	0.26	0.99 [0.96, 1.02]	0.58	0.17 [0.081, 0.26]	<0.001

Result of the multivariate logistic regression predicting the binary outcome “became AAA1 IgG positive” or “became AF3L1 IgG positive” and of the multivariate linear regression predicting the optical density (OD) change of anti-ApoA-1 (AAA1) IgG and anti-F3L1 (AF3L1) IgG between sample 1 and sample 2, as a function of the anti-S1 positivity, the sex, the age of the patient, the disease, the years of biological disease-modifying antirheumatic drug (bDMARD) treatment between sample 1 and sample 2, the cumulated glucocorticoid (GC) dose between sample 1 and sample 2, the delay between sample 1 and sample 2, and the disease duration. RA, rheumatoid arthritis; AxSpa, axial spondyloarthritis; PsA, psoriatic arthritis; bDMARDs, biologic disease-modifying antirheumatic drugs; GC, glucocorticoids.

## Discussion

The main findings of this study is that COVID-19 induces a humoral response against the c-terminal part apoA-1 in IRD, corroborating and extending previous observations in immunocompetent populations (10, 11). Furthermore, the significant association observed between anti-S1 and AF3L1 responses is also in line with previous studies, suggesting that the polyclonal AAA1 autoantibody response in humans is preferentially orientated against the c-terminal alpha helical part of the protein (14, 15). The rationale for this finding is a linear sequence homology between the Spike protein of SARS-CoV2 and the c-ter region of ApoA-1, which may underpin the production of AAA1 in SARS-CoV2-affected individuals (10). The reason why such associations could not be reproduced for autoantibodies against the full ApoA-1 molecule despite the trend is most likely due to the low statistical power of this study, although other explanations cannot be formally excluded.

Given the known association between this type of humoral autoimmune response and poor evolution of the disease, this result is an additional argument in favour of vaccination against Sars-CoV2. The fact that IRD shares risk factors with COVID-19 and that immunomodulatory therapies tend to increase the risk of poor COVID-19 outcomes were already strong arguments in favour of vaccination (16, 17). However, due to the potential impact of immunomodulatory therapies on vaccine efficacy and safety, it is important to IRD patients to discuss their vaccination options with their healthcare provider, who can provide personalised recommendations based on their patient’s individual medical history, current medications, and overall health status (18).

The second notable finding of this study is that the AAA1 and AF3L1 seroprevalences were evenly distributed across the whole IRD spectrum of diseases. Due to the proinflammatory biological properties of AAA1, and their established association with poorer CV and general outcomes, further studies are warranted to determine the clinical relevance of such autoimmune biological signature in IRD other than RA.

The strengths of this study are a representative sample of IRD patients followed and sampled before and during the pre-/post-pandemic period. The fact that almost all sampling occurred before the start of the vaccination campaign in Switzerland ensures that the anti-S1 seropositivity measured is caused by SARS-CoV2 infections and not by vaccinations.

There are several limitations to the present study. The first resides in the low number of anti-S1-positive patients in our cohort, probably related to the relatively short 8-month follow-up after the beginning of the SARS-CoV2 pandemic and the low fraction of IRD patients exposed to SARS-CoV2 in this high-risk population. This in turn prevented us to explore the possible associations with CV complications or long COVID syndrome, as previously reported (5, 11, 12), which is an important limitation of the present study. Thirdly, although we took care to minimise the impact of possible confounding factors in our adjusted analyses, we cannot formally rule out that unmeasured confounding factors could have blunted the present results. Finally, we limited our analyses to AAA1 autoantibodies and did not consider other autoantibodies, such as rheumatoid factors (RF), anti-citrullinated protein antibodies (ACPA), or anti-HDL antibodies known to be associated with RA. ACPA increase and RA flaring have been previously described after SARS-CoV2 infection, but it is currently still unclear if this finding is causal or a spurious finding (19). Future work is warranted to replicate and validate these preliminary results in larger cohorts with a longer follow-up duration.

In conclusion, this report reveals that in a subset of IRD patients exposed to a SARS-CoV2 infection, humoral autoimmune response against the c-terminal region of ApoA-1 can occur, extending the concept that exposure to infectious agents may lead to the development of autoimmunity in IRD patients. Because SARS-CoV2 infections are still expanding, the incidence of AAA1 seropositivity is expected to increase overall, including in IRD patients. The clinical implications of these biomarkers are still unclear and remain to be addressed.

## Patient and public involvement statement

This research was done without patient involvement. Patients were not invited to comment on the study design and were not consulted to develop patient relevant outcomes or interpret the results. Patients were not invited to contribute to the writing or editing of this document for readability or accuracy.

## Data availability statement

The raw data supporting the conclusions of this article will be made available by the authors, without undue reservation.

## Ethics statement

The studies involving human participants were reviewed and approved by the local ethics committee of the University Hospital of Geneva (PB_2018-00317). The patients/participants provided their written informed consent to participate in this study.

## Author contributions

All the authors have provided substantial contributions to the conception or design of the work, the acquisition of the data, and the interpretation of data. DM performed the data management and the statistical analysis. CL and DM made the first draft. All the other authors participated in the final drafting of the work or revising it critically for important intellectual content. All authors contributed to the article and approved the submitted version.

## References

[1] Satta N Frias MA Vuilleumier N Pagano S. Humoral immunity against HDL particle: a new perspective in cardiovascular diseases? Curr Pharm Des (2019) 25(29):3128–46. doi: 10.2174/1381612825666190830164917 31470782

[2] Croca S Bassett P Chambers S Davari M Alber KF Leach O. IgG anti-apolipoprotein a-1 antibodies in patients with systemic lupus erythematosus are associated with disease activity and corticosteroid therapy: an observational study. Arthritis Res Ther (2015) 17(1):26. doi: 10.1186/s13075-015-0539-z 25890187 PMC4354753

[3] Dinu AR Merrill JT Shen C Antonov IV Myones BL Lahita RG. Frequency of antibodies to the cholesterol transport protein apolipoprotein A1 in patients with SLE. Lupus (1998) 7(5):355–60. doi: 10.1191/096120398678920262 9696140

[4] Antiochos P Marques-Vidal P Virzi J Pagano S Satta N Bastardot F. Association between anti-apolipoprotein a-1 antibodies and cardiovascular disease in the general population. Results CoLaus study. Thromb Haemost. (2016) 116(4):764–71. doi: 10.1161/ATVBAHA.117.309602 27384400

[5] Pagano S Magenta A D'Agostino M Martino F Barilla F Satta N. Anti-ApoA-1 IgGs in familial hypercholesterolemia display paradoxical associations with lipid profile and promote foam cell formation. J Clin Med (2019) 8(12):2035. doi: 10.3390/jcm8122035 31766415 PMC6947407

[6] Smatti MK Cyprian FS Nasrallah GK Al Thani AA Almishal RO Yassine HM. Viruses and autoimmunity: a review on the potential interaction and molecular mechanisms. Viruses (2019) 11(8):762. doi: 10.3390/v11080762 31430946 PMC6723519

[7] Dotan A Muller S Kanduc D David P Halpert G Shoenfeld Y. The SARS-CoV-2 as an instrumental trigger of autoimmunity. Autoimmun Rev (2021) 20(4):102792. doi: 10.1016/j.autrev.2021.102792 33610751 PMC7892316

[8] Kanduc D. From anti-SARS-CoV-2 immune responses to COVID-19 via molecular mimicry. Antibodies (Basel) (2020) 9(3):33. doi: 10.3390/antib9030033 32708525 PMC7551747

[9] Grifoni A Sidney J Zhang Y Scheuermann RH Peters B Sette A. A sequence homology and bioinformatic approach can predict candidate targets for immune responses to SARS-CoV-2. Cell Host Microbe (2020) 27(4):671–80 e2. doi: 10.1016/j.chom.2020.03.002 32183941 PMC7142693

[10] Pagano S Yerly S Meyer B Juillard C Suh N Le Terrier C. SARS-CoV-2 infection as a trigger of humoral response against apolipoprotein a-1. Eur J Clin Invest. (2021) 51(11):e13661. doi: 10.1111/eci.13661 34324704 PMC8420318

[11] L'Huillier AG Pagano S Baggio S Meyer B Andrey DO Nehme M. Autoantibodies against apolipoprotein a-1 after COVID-19 predict symptoms persistence. Eur J Clin Invest. (2022) 52(10):e13818. doi: 10.1111/eci.13818 35598178 PMC9348059

[12] Vuilleumier N Bas S Pagano S Montecucco F Guerne PA Finckh A. Anti-apolipoprotein a-1 IgG predicts major cardiovascular events in patients with rheumatoid arthritis. Arthritis Rheumatol (2010) 62(9):2640–50. doi: 10.1002/art.27546 20506304

[13] Antiochos P Marques-Vidal P Virzi J Pagano S Satta N Hartley O. Impact of CD14 polymorphisms on anti-apolipoprotein a-1 IgG-related coronary artery disease prediction in the general population. Arterioscler Thromb Vasc Biol (2017) 37(12):2342–9. doi: 10.1161/ATVBAHA.117.309602 29074586

[14] Vuilleumier N Antiochos P Marques-Vidal P Pagano S Virzi J Satta N. Prognostic and therapeutic considerations of antibodies against c-ter apolipoprotein a-1 in the general population. Clin Transl Immunol (2020) 9(12):e1220. doi: 10.1002/cti2.1220 PMC7734471 33343896

[15] Teixeira PC Ducret A Ferber P Gaertner H Hartley O Pagano S. Definition of human apolipoprotein a-I epitopes recognized by autoantibodies present in patients with cardiovascular diseases. J Biol Chem (2014) 289(41):28249–59. doi: 10.1074/jbc.M114.589002 PMC4192480 25170076

[16] Murdaca G Noberasco G Olobardi D Lunardi C Maule M Delfino L. Current take on systemic sclerosis patients' vaccination recommendations. Vaccines (Basel) (2021) 9(12):1426. doi: 10.3390/vaccines9121426 34960174 PMC8708328

[17] Strangfeld A Schafer M Gianfrancesco MA Lawson-Tovey S Liew JW Ljung L. Factors associated with COVID-19-related death in people with rheumatic diseases: results from the COVID-19 global rheumatology alliance physician-reported registry. Ann Rheum Dis (2021) 80(7):930–42. doi: 10.1136/annrheumdis-2020-219498 PMC7843211 33504483

[18] Bijlsma JW Force EC-T. EULAR 2021 updated viewpoints on SARS-CoV-2 vaccination in patients with RMDs: a guidance to answer patients' questions. Ann Rheum Dis (2022) 81(6):786–8. doi: 10.1136/annrheumdis-2021-221965 35121591

[19] Derksen V Kissel T Lamers-Karnebeek FBG van der Bijl AE Venhuizen AC Huizinga TWJ. Onset of rheumatoid arthritis after COVID-19: coincidence or connected? Ann Rheum Dis (2021) 80(8):1096–8. doi: 10.1136/annrheumdis-2021-219859 33648960

